# Multicentric infantile myofibromatosis with extensive visceral involvement in a newborn: case report

**DOI:** 10.1186/s13052-025-02055-y

**Published:** 2025-07-08

**Authors:** Rossella Vitale, Manuela Capozza, Antonia Filannino, Michele Quercia, Chiara Novielli, Grazia Calderoni, Francesco De Leonardis, Nicola Santoro, Stefania Martino, Nicoletta Resta, Nicola Laforgia

**Affiliations:** 1 Department of Interdisciplinary Medicine, Neonatology and NICU, University of Bari “Aldo Moro”, Bari, 70124 Italy; 2 Pediatric Oncology AOUC of Policlinico of Bari, Bari, 70124 Italy; 3 Medical Genetics Unit, Department of Precision and Regenerative Medicineand, Ionian Area University of Bari “Aldo Moro”, Bari, 70124 Italy

**Keywords:** Infantile myofibromatosis, Case report, Fibrous tumor, Visceral involvement, Watch and wait

## Abstract

**Background:**

Infantile myofibromatosis, a rare soft tissue neoplasm that may present at birth or in early infancy, is the most common fibrous tumor of infancy and early childhood. Diagnosis could be challenging due to different clinical presentation. Very few cases are detected prenatally and visceral involvement is extremely rare.

**Case presentation:**

We present a case of Disseminated Infantile Myofibromatosis with challenging prenatal ultrasound and misleading clinical presentation. Diagnosis was very difficult and confirmed by pathology results obtained after birth.

**Conclusions:**

Visceral involvement constitutes a specific unfavorable prognostic factor but a watchful waiting approach would always be appropriate, since spontaneous regression and a favourable evolution are possible and age-related chemotherapy severe side effects and long-term sequelae are matter of concern.

## Background

Infantile myofbromatosis (IM) is a mesenchymal disorder characterized by nodules in the skin, muscle, bone, and, more rarely, in visceral organs. Although rare, IM is the most common fibrous tumor of infancy and early childhood, with an incidence of 1: 150,000 [1].

IM can present as a solitary form (SFIM), multicentric form without visceral involvement (MFIM) or disseminated form with visceral involvement (DFIM) [2]. Prognosis of SFIM and MFIM are usually good, while the mortality rate of DFIM is up to 73% [3]. Although spontaneous tumoral regression is frequent, progression or recurrence are also possible, so individual follow-up is mandatory.

## Case presentation

E.E. is a male full term newborn (38 weeks), small for gestational age (SGA), born by emergency caesarean section because of alterations of cardiotocographic monitoring (CTG) in an uneventful pregnancy. APGAR 8-9.

The ultrasound evaluation in the third semester showed cysts in different regions of the body, with the bigger one (3 cm of diameter) into the right maxillary bone, but other similar formations were found in both legs and paravertebral muscles. No further investigation has been done during pregnancy.

**Fig. 1:**
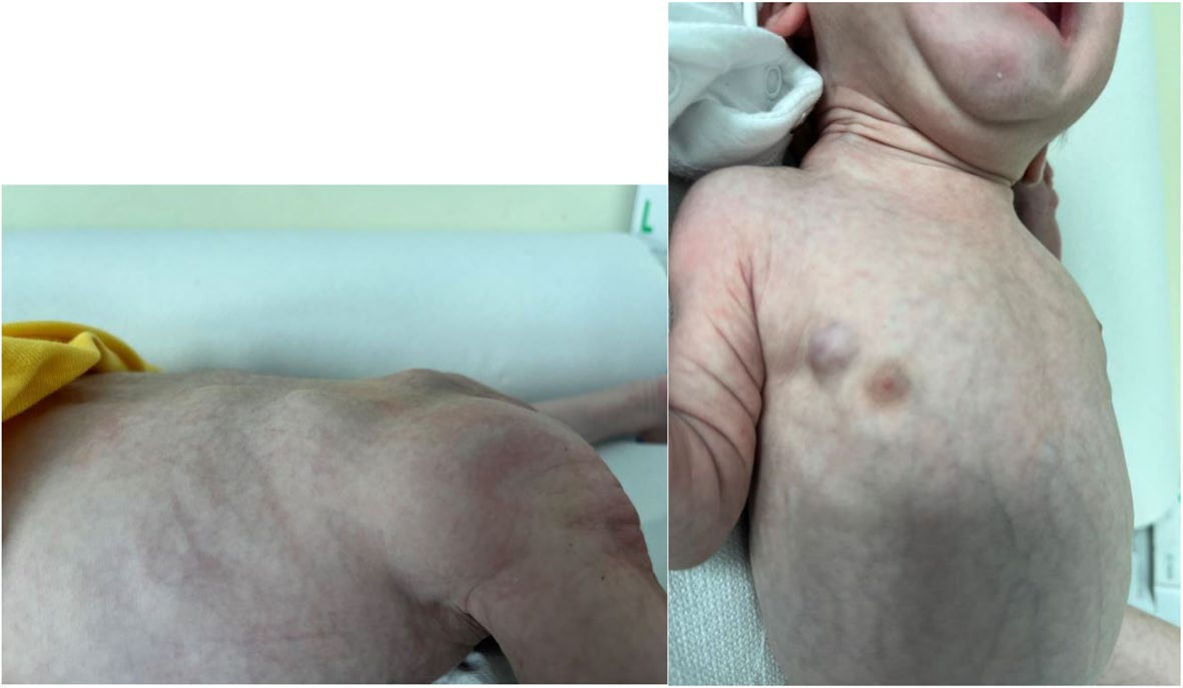
First physical examination: At birth physical examination showed the presence of firm non-tender soft tissue masses in the right maxillary bone, in both legs and paravertebral muscles. Also a blue-berry like nodule was evident in the periareolar right region

At birth physical examination confirmed the presence of firm non-tender soft tissue masses in the same regions described in prenatal period and other similar lesions were present in lower back region, arms and neck. Also a blue-berry like nodule was evident in the periareolar right region (Fig. 1).

No abdominal masses were palpable and the rest of the exam was normal.

In the second day of life, hyporeactivity, axial hypotonia and horizontal nystagmus were detected. Hyperammonemia, metabolic alkalosis and hypoalbuminemia were found and he was admitted to our Neonatal Intensive Care Unit.

Enteral feeding was immediately stopped and administration of albumin, carglumic acid and adequate parenteral nutrition with low protein and carbohydrate intake was started.

Abdominal ultrasound showed multiple hepatic hypoechoic formations (d max = 6 mm) and abdominal effusion; brain ultrasound and echocardiography were normal, as well as newborn metabolic screening and plasmatic and urinary amino acid.

In the following days, general conditions improved and enteral feeding was started.

**Fig. 2:**
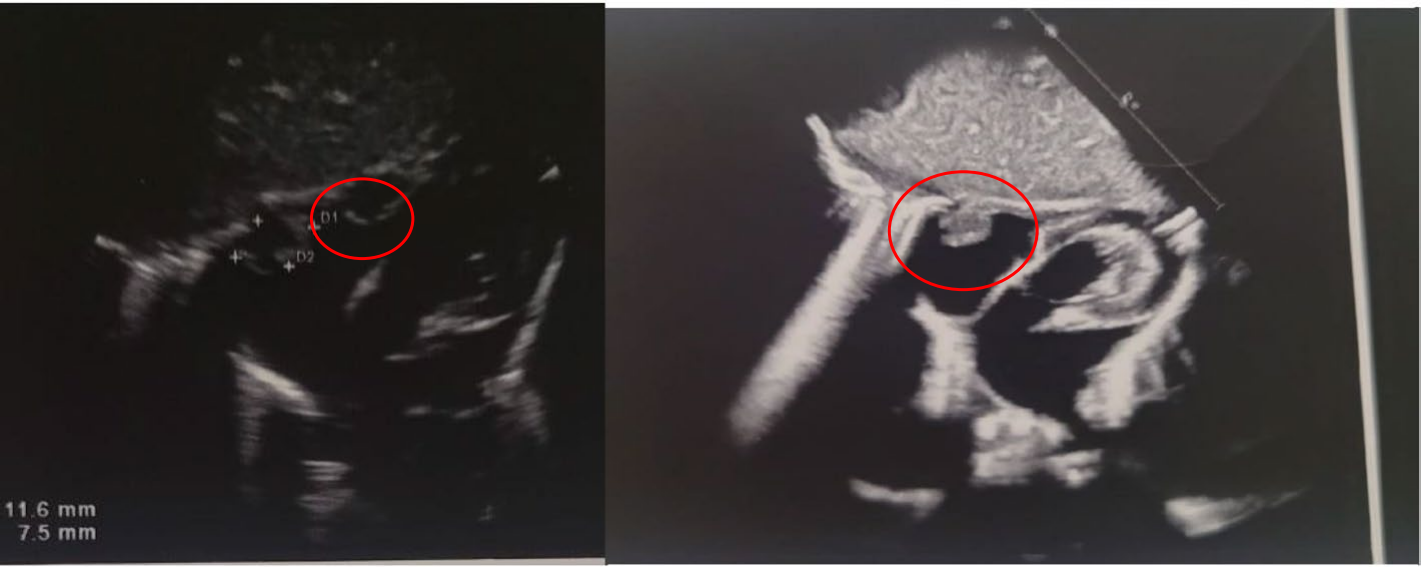
Echocardiogram: A hyperechoic formation with regular edges (1.5 × 1.5 cm) was found in the right atrium

In 12° day of life, because of a heart murmur echocardiogram was performed and a hyperechoic formation with regular edges (1.5 × 1.5 cm) was found in the right atrium (Fig. 2).

**Fig. 3:**
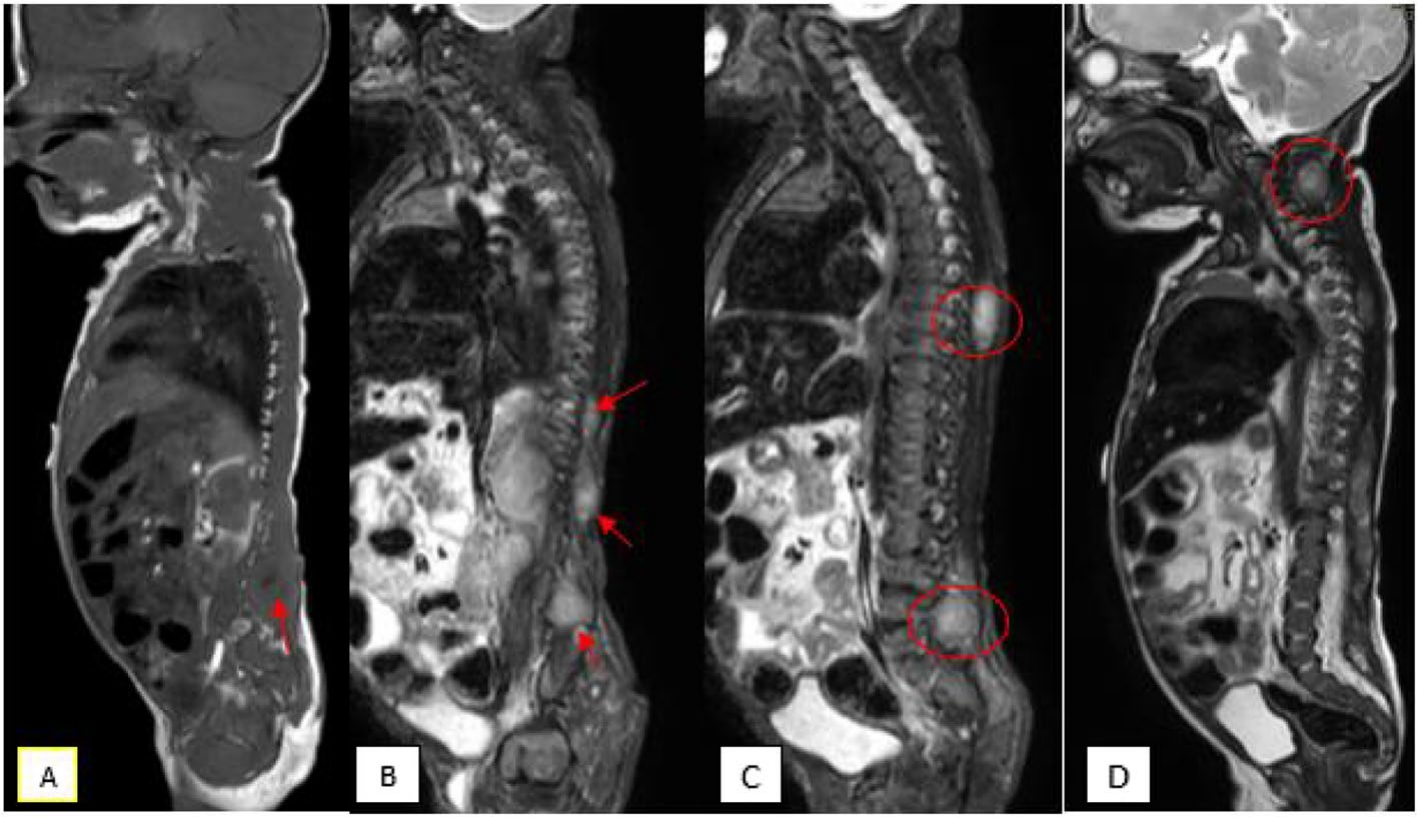
MRI at the age of 20 days: Paraspinal lesions demonstrated inhomogeneous T2 hypointensity and T1 isointensity with contrast enhancement of 10 scattered lesions in dorsal and paravertebral region

**Fig. 4:**
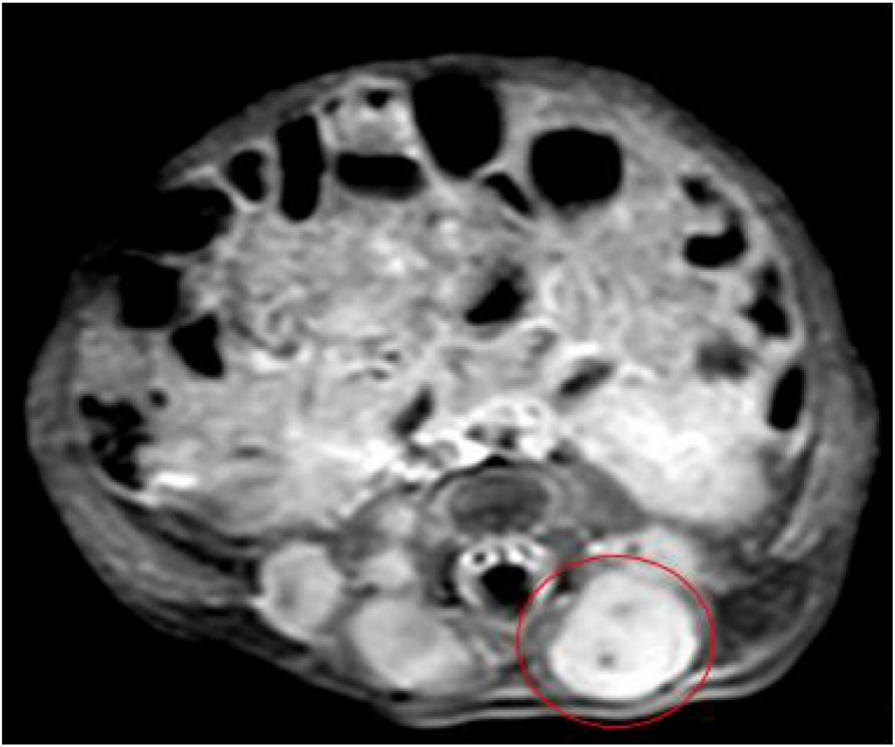
MRI at the age of 20 days: Right sacral lesion in axial T1 with contrast enhancement

**Fig. 5:**
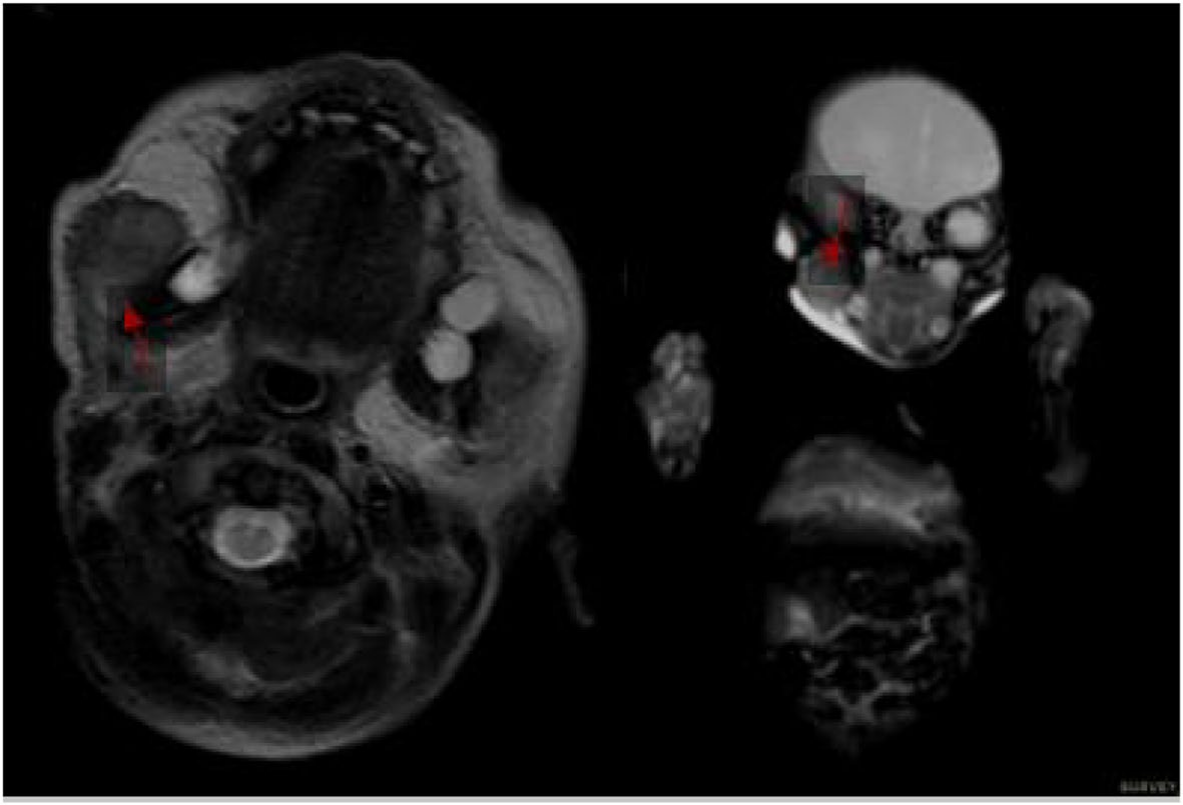
MRI at the age of 20 days: Mandibolar right formation on axial T1 and survey coronal

MRI at the age of 20 days demonstrated inhomogeneous T2 hypointensity and T1 isointensity with contrast enhancement of 10 scattered lesions in dorsal and paravertebral region but also revealed multiple additional tumor foci in the muscles of all four extremities, left cerebellar hemisphere and liver lobes with the same imaging characteristics (Figs. 3,4,5).

Biopsy of the paraspinal bigger lesion showed mesenchymal spindle cell proliferation. On immunohistochemical analysis, tumor cells stained strongly positive for SMA (Smooth Muscle Autoantibodies) but negative for CD34 and desmin. The Ki67 index was only 3%, sign of low proliferative activity.

Genomic DNA (gDNA) was extracted from the proband and parents’ peripheral blood (PB) samples for molecular analyses using next-generation sequencing (NGS). Trio-based clinical exome sequencing (CES) was performed utilizing the Trusight One Expended Sequencing Panel Kit (Illumina, San Diego, CA, USA). Libraries were prepared following the NextEra Flex for Enrichment protocol (Illumina) and sequenced on the NextSeq550Dx Illumina Platform (Illumina). Sequencing data were analyzed using the NextSeq control software v4.2.0 and Local Run Manager software v. 4.0.0, both provided by Illumina.

Variant calling data were analyzed using Geneyx analysis software v 5.15 (Geneyx, Herzliya,Israel), following reads alignment against the human genome reference (GRCh37) by the BWA Aligner software v. 11. [4] Filtering and prioritization of variants were performed using HPO terms [5]: “Abnormality of the airline”, “Long eyebrows and eyelashes”, “retrognathia”, “nodules hard consistency”, “Bone cyst”, “spinal cord lesion” and “skin nodule”. BAM (Binary Alignment Map) files were visually inspected by IGV (Integrative Genome Viewer) software 2.16.0 and Alamut Visual Plus Genome Viewer (Sophia Genetics. Lausanne, Switzerland). Variants were annotated according to the Human Genome Variants Society (HGVS) recommendations [6] and classified using interpretation tools (Varsome, Franklin by Genoox), public databases (ClinVar, LOVD, OMIM) and American College of Medical Genetics and Genomics (ACMG) criteria [7].

Trio-based CES analyses identified a heterozygous state variant, paternally inherited, in the intron 21 of the gene PDGFRB (NM_002609.4): c. 2905-8G > A; p.? (rs201866603).

A multidisciplinary counselling was started with paediatric oncologists, to evaluate the possibility of starting a pharmacological treatment, according to literature.

Because of the low weight of our patient (2380 g) and also the possibility of spontaneous regression no therapy was started and he was discharged with a close follow-up.

**Fig. 6:**
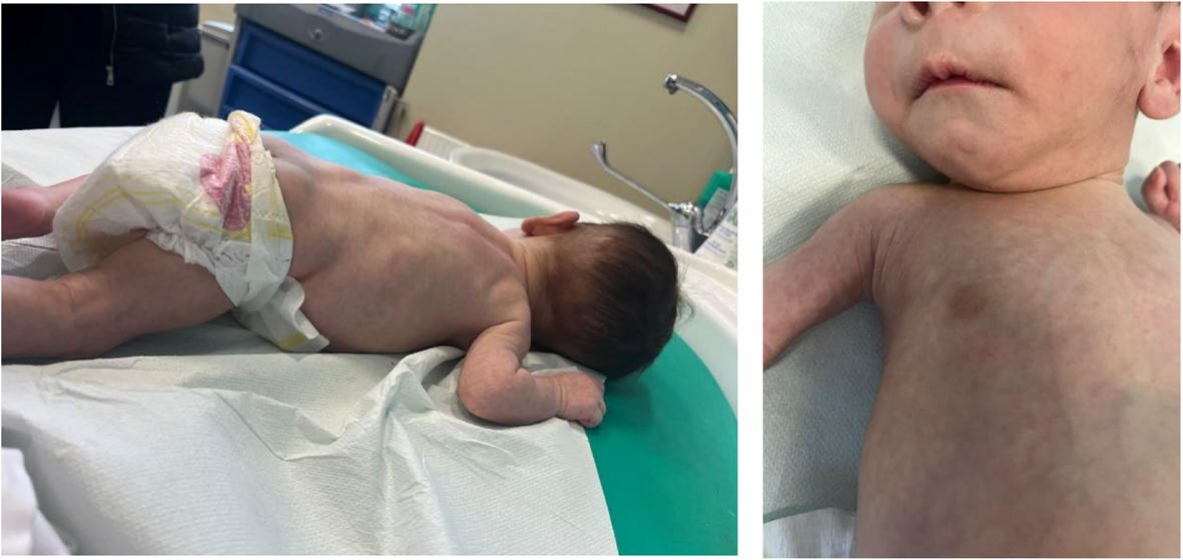
Six-months examination: Progressive regression of the lesions

**Fig. 7:**
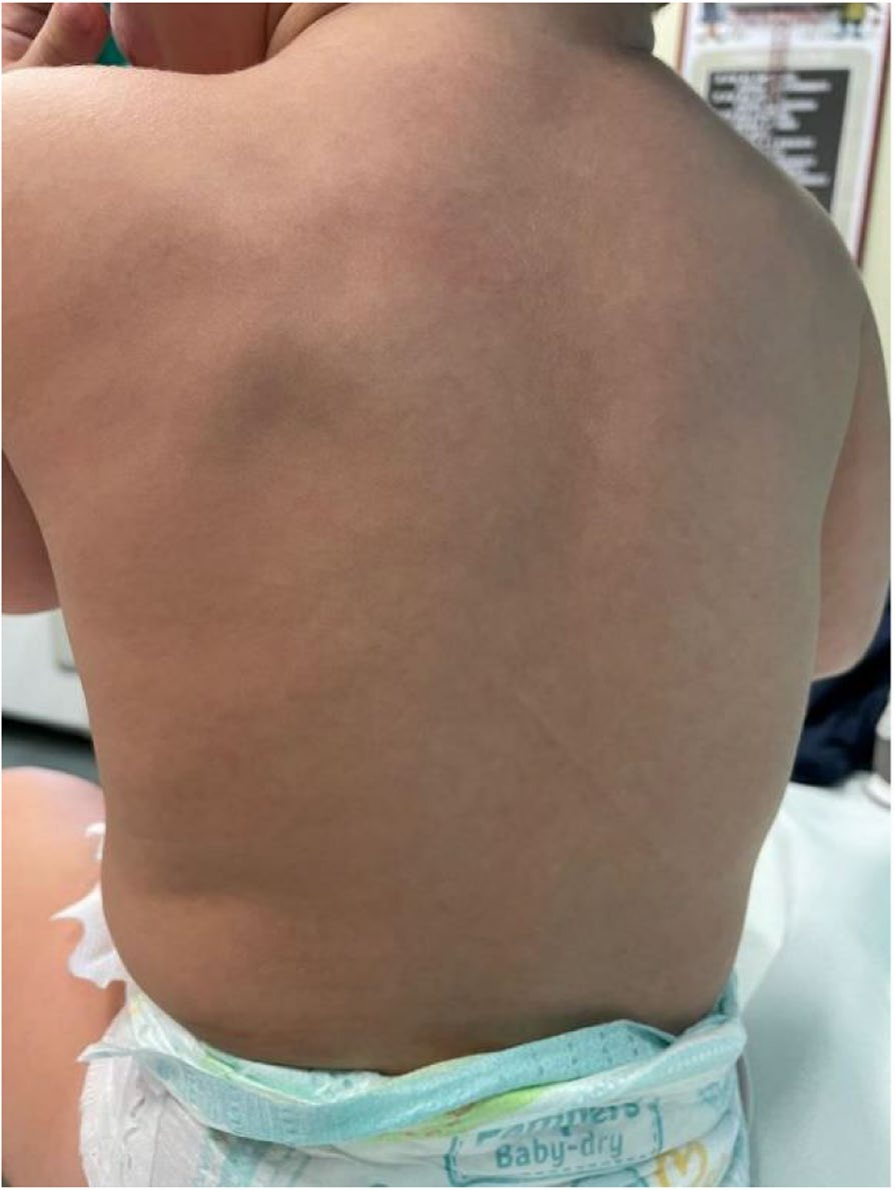
One-year examination: At 1-year he showed good growth with complete disappearance of all the lesions

During the first year of life he showed good growth, adequate neurocognitive development, and progressive regression of all the lesions at 6 months (Fig. 6) up to the complete disappearance (Fig. 7) at 1-year except for the cardiac one.

In the right atrium, echocardiography highlighted an echogenic formation of 1 × 1 cm with regular contours which starts from the lateral wall and affects the plane of the tricuspid but which does not obstruct the right inflow and outflow.

## Discussion and conclusions

The incidence of soft tissue fibrous tumours in newborns is about 1/15000 and 35% of them are IM [8].

Characteristic pathologic findings include generally: (1) not uniform size; (2) gray-white in color; (3) the necrosis and cystic change in the middle of nodules by the naked eyes. The characteristic microscope examination of tumor cells includes: (1) myofibroblastic cells arranged in short bundles or swirls; (2) spindle shape of myofibroblastic cells; (3) seldom mitosis of nucleus; (4) necrosis in the tumor and calcification in the mesenchymal tissue; and (5) some tumor cells growing under the vascular endothelium [9].

Pathogenic or likely pathogenic variants in the *PDGFRB* gene (MIM * 173,410, PLATELET-DERIVED GROWTH FACTOR RECEPTOR, BETA) are linked to various clinical conditions inherited in an autosomal dominant pattern, including Myofibromatosis, infantile, 1 (OMIM # 228550). To date, over 600 PDGFRB variants have been reported in the ClinVar database (last accessed 27 November 2024), of which approximately 36 variants have been classified as pathogenic or likely pathogenic. Most of these variants are missense and inframe indels located within the kinase domain of the PDGFRB protein. Germline deleterious variants associated with familial myofibromatosis are known to constitutively activate PDGFRB signaling, indicating a gain-of-function disease mechanism [10, 11].

The identified variant is listed in the gnomAD database, with a maximum subpopulation frequency of 0.0065% in the European (non-Finnish) population (5-149,497,421-C-T, gnomAD v2.1.1, gnomad.broadinstitute.com). ClinVar also contains an entry for this variant (Variation ID: 1486244). SpliceAI, an silico tool for predicting the effect of sequence changes on RNA splicing suggests that this variant may disrupt the consensus splice site (SpliceAI AG: 1.00, splice altering strong). The variant has not been previously reported in the literature or observed in individuals with clinical features of PDGFRB-related conditions. Based on ACMG guidelines (criteria PM2, PP3), it has been classified as a Variant of Uncertain Significance (VUS).

Most cases occur spontaneously, but familial forms associated with germline mutations of PDGFRB are described, mainly in the multicentric form of the disease and genetic analysis may be helpful in patient’s treatment.

Familial IM follows an autosomal dominant mode of inheritance and is linked to PDGFRB germline variants. Somatic PDGFRB variants were also detected in solitary and multifocal IM lesions. PDGFRB variants associated with IM constitutively activate PDGFRB kinase activity in the absence of its ligand. Germline variants have lower activating capabilities than somatic variants and, thus, require a second cis-acting hit for full receptor activation. Typically, these mutant receptors remain sensitive to tyrosine kinase inhibitors such as imatinib. The SIOPE Host Genome Working Group, consisting of pediatric oncologists, clinical geneticists and scientists, met in January 2020 to discuss recommendations for genetic testing and surveillance for patients who are diagnosed with IM or have a family history of IM/PDGFRB germline variants [12].

The prognosis for patients with localized disease (LD), where the tumors are confined to the skin, muscle, or bone, is generally excellent. A study involving 71 patients with LD reported a 5-year overall survival rate of 95% (± 6%), indicating a high likelihood of long-term survival in these cases. Similarly, patients with multifocal disease (MFD) but without visceral involvement exhibit a comparable prognosis, with the same study reporting a 5-year overall survival rate of 95% (± 10%) [13].

However, the prognosis worsens significantly when the disease affects vital organs. Cases of multifocal disease with visceral involvement are associated with a much higher risk of mortality, with reported rates ranging from 33 to 76%, primarily due to complications affecting the cardiopulmonary and gastrointestinal systems [14, 15].

A key characteristic of IM is its potential for spontaneous regression, particularly in cases without visceral involvement. Many lesions undergo natural resolution within one to two years after diagnosis [16].

While many IM lesions regress on their own, there is a possibility of recurrence, particularly in cases where the initial tumors had visceral involvement or were only partially removed [17].

Functional impairments may also arise depending on the tumor's location. For example, tumors in the mandibular region can lead to dental and speech issues, especially if they interfere with bone development [18].

Since many of these lesions tend to regress over time, as seen in different case reports [19, 20] the traditional approach is to observe quiescent lesions, while growing myofibromas are treated with surgery or chemotherapy.

The "watch and wait" approach following biopsy has demonstrated success in numerous cases of both solitary lesions and visceral involvement. This was evidenced by Sparber et al., who analyzed 95 patients with IM enrolled in five Cooperative Weichteilsarkom Studiengruppe (CWS) trials. Specifically, the W&W strategy was implemented in 55 out of 71 patients with primary localized disease, resulting in complete remission in 36 of them. Additionally, it was applied to seven out of 12 patients with visceral involvement. The authors concluded that a strict "watch and wait" strategy was appropriate for most patients [21].

Treatment decisions are based on the neoplasms' location and symptom profile, with solitary IM typically requiring minimal intervention. In contrast, multicentric IM and generalized IM with visceral involvement may necessitate more aggressive treatment when vital organs are affected.

In neonates and infants, however, either surgical removal of large, infiltrative masses or chemotherapy is associated with high morbidity [22]. Several chemotherapy regimens have been investigated, including combinations of vinblastine and methotrexate, vincristine and dactinomycin, and cyclophosphamide [9, 23, 24].

However, it remains uncertain whether these chemotherapy options definitively treat myofibromas or if the lesions would resolve spontaneously regardless of treatment. Sparber et al. also observed that systemic therapy with MTX/VBL remains an option in cases of progressive disease (PD), however, chemotherapy failed to salvage three patients with PD, including one with a secondary rhabdoid tumor, highlighting the urgent need for new treatment options.

Recently, tyrosine kinase inhibitors (TKIs), such as imatinib and sunitinib, have emerged as effective treatment options for both autosomal dominant and somatic cases of IM with PDGFRB mutations [12, 25–27]. Imatinib offers a new and effective therapy for selected IM patients by targeting their known activating PDGFRB variants. In vitro studies suggest that PDGFRB activating mutations associated with familial IM and severe overgrowth syndrome, are sensitive to tyrosine kinase inhibitors such as imatinib, nilotinib and ponatinib [11].

Preliminary phase clinical trials using sorafenib-containing regimens in children have shown potential antitumor activity [28].

The role of these agents in treating IM remains largely unclear. In literature there are 3 different case reports to document the effective use of tyrosine kinase inhibition in treating a patient with IM; in one case, a patient with the previously identified 1681 C > A missense heterozygous germline mutation and refractory IM was successfully treated with sunitinib and vinblastine [27].

In another case, a patient with multicentric IM harboring a germ-line PDGFRB p.N666H mutation responded positively to imatinib treatment [29].

Another recent case report describes a unique condition in which cutaneous and pulmonary nodules progressed despite treatment with several chemotherapy regimens—including vinblastine and methotrexate, VAC, and 2-CDA—but responded rapidly to two different tyrosine kinase inhibitors [30].

However, possible long-term effects in this “benign” disease should be considered carefully when using new agent drugs in infants and children, nonetheless, TKI-associated growth impairment is a significant concern, particularly for prolonged treatment durations. Consequently, TKIs are generally reserved for severe cases involving vital organs and further research is needed to develop optimal protocols for tapering and discontinuing TKIs in the management of IM.

A case of disseminated visceral infantile myofibromatosis with first clinical appearance misleading for metabolic disease due to the liver involvement has been presented.

MRI was necessary to better define the dissemination of the lesions and biopsy confirmed the diagnosis.

Even though visceral involvement in DFIM is deemed as a specific poor prognostic factor, we confirm that a watchful waiting approach avoiding front-line active treatments should be always considered, since a spontaneous regression and a favourable evolution is possible and age-related chemotherapy severe side effects and long-term sequelae are matter of concern.

IM remains largely enigmatic, displaying a typically benign course that can, on rare occasions, lead to devastating outcomes; our experience suggests that the cautious use of novel drugs should be reserved for the most severe cases, Particular attention should be given to neonates, who are more likely to develop collateral effects. In PDGFRB-mutated IM, imatinib and sunitinib may represent promising future therapies. Nevertheless, given the generally benign nature of this disease, the potential long-term effects of novel agents in infants and children must be carefully considered and future research should focus on identifying minimally invasive therapies and establishing a standardized observation protocol, which is currently lacking.

## Data Availability

Data will be made available on reasonable request.

## Abbreviations

IM: Infantile myofbromatosis
SFIM: Solitary form
MFIM: Multicentric form without visceral involvement
DFIM: Disseminated form with visceral involvement
CTG: Cardiotocographic monitoring
MRI: Magnetic resonance imaging
SMA: Smooth muscle autoantibodies
Gdna: Genomic DNA
PB: Peripheral blood
NCS: Next-generation sequencing
CES: Trio-based clinical exome sequencing
SGA: Small for gestational age
LD: Localized disease
W&W: Watch and wait
